# Novel Techniques of Sperm Selection for Improving IVF and ICSI Outcomes

**DOI:** 10.3389/fcell.2019.00298

**Published:** 2019-11-29

**Authors:** Iván Oseguera-López, Sara Ruiz-Díaz, Priscila Ramos-Ibeas, Serafín Pérez-Cerezales

**Affiliations:** ^1^Unidad Iztapalapa, Universidad Autónoma Metropolitana, Mexico City, Mexico; ^2^Mistral Fertility Clinics S.L., Clínica Tambre, Madrid, Spain; ^3^Department of Animal Reproduction, Instituto Nacional de Investigación y Tecnología Agraria y Alimentaria, Madrid, Spain

**Keywords:** sperm selection, ICSI, IVF, ARTs, sperm quality

## Abstract

Almost 50% of the infertility cases are due to male factors. Assisted reproductive technologies (ARTs) allow to overcome the incapacity of these patients’ spermatozoa to fertilize the oocyte and produce a viable and healthy offspring, but the efficiency of the different techniques has still the potential to improve. According to the latest reports of the European Society of Human Reproduction and Embryology (ESHRE) and the Centers for Disease Control and Prevention of the United States (CDC), the percentages of deliveries per ART cycle in 2014 and 2016 were 21 and 22%, respectively. Among the reasons for this relatively low efficiency, the quality of the spermatozoa has been pointed out as critical, and the presence of high percentages of DNA-damaged spermatozoa in patients’ ejaculates is possibly one of the main factors reducing the ARTs outcomes. Thus, one of the main challenges in reproductive medicine is to ensure the highest quality of the spermatozoa used in ARTs, and specifically, in terms of genetic integrity. The latest techniques for the preparation and selection of human spermatozoa are herein discussed focusing on those proven to improve one or several of the following parameters: sperm genetic integrity, fertilization capacity, embryo production, and *in vitro* survival, as well as pregnancy and delivery rates following *in vitro* fertilization (IVF) and intracytoplasmic sperm injection (ICSI). In addition, we discuss the potential of techniques developed in non-human mammals that could be further transferred to the clinic.

## Introduction

### Current Efficiency of ARTs

According to the World Health Organization, infertility is defined as “a disease characterized by the failure to establish a clinical pregnancy after 12 months of regular, unprotected sexual intercourse or due to an impairment of a person’s capacity to reproduce either as an individual or with his/her partner” (Zegers-Hochschild et al., 2017). Around 15–20% of couples within the reproductive age are infertile and in ∼50% of the cases, the male factor is present. Furthermore, in 30–40% of cases, the male infertility is idiopathic (Katz et al., 2017). In order to overcome infertility, a number of clinical treatments have been successfully developed and are englobed in what is known as assisted reproductive technologies (ARTs). These involve *in vitro* handling of oocytes, sperm, and embryos for their use in reproduction and comprise *in vitro* fertilization (IVF), embryo transfer, intracytoplasmic sperm injection (ICSI), embryo biopsy, preimplantation genetic testing, and gamete and embryo cryopreservation. The use of ARTs has been steadily rising in the developed countries in last decades (Ferraretti et al., 2017) and from 1997 to 2014, at least 1.5 million babies have been conceived by these techniques in Europe (De Geyter et al., 2018). ARTs are also used in the veterinarian clinical practice and livestock production. However, assisted insemination (AI) is still the preferential reproductive procedure in breeding programs because of its simplicity and effectiveness (Hansen, 2014; Knox, 2016). In cattle embryo production, because of its high efficiency, IVF is intensively used in routine breeding management and millions of calves have been born by this technique (Sirard, 2018). In contrast, ARTs are not applied in sheep and goat farming although the technology has already been developed (Paramio and Izquierdo, 2014). In porcine, the main disadvantage when producing embryos by IVF is the high level of polyspermy (Nagai et al., 2006). This problem can be bypassed by ICSI since normal blastocysts can be produced by this technique with the same efficiency than IVF (Wu et al., 2001). In livestock species, ICSI is not used to overcome male infertility unless the male has a high value, as for endangered species or stallion (Galli et al., 2014; Herrick, 2019), because of the low efficiencies of embryo production achieved in farm animals with this technique (Salamone et al., 2017). In stallion, only two foals have been reported to born following IVF using *in vivo* matured oocytes. As a result in this species, ICSI is the only alternative and has allowed the production of foals from *in vitro* matured oocytes mainly in the clinical management of infertile individuals (Galli et al., 2014).

Despite the wide use of ARTs in the last decades, their efficiency still has the potential to improve. Using both techniques (ICSI or IVF), the latest efficiencies reported by the American and European societies of reproduction and fertility are around 37% of pregnancies and 25% of deliveries per embryo transfer (CDC, 2018; De Geyter et al., 2018). These percentages were higher when fresh oocytes from donors were used, reporting 54 and 38% of pregnancies and deliveries per embryo transfer, respectively. In livestock species, ARTs and specially ICSI are also inefficient (García-Roselló et al., 2009; Salamone et al., 2017) or have not been successfully developed, as in the case of IVF in horse (Leemans et al., 2016) and ICSI in cattle (Li et al., 2004). These low efficiencies can be due to multiple factors such as suboptimal *in vitro* conditions, injuries associated to gametes and embryo manipulation, subjacent male and female factors, etc. We overview herein the different methods recently developed in order to improve ARTs, focusing on the strategy of spermatozoa selection.

### Why It Is Important to Select the Spermatozoa? Semen Quality and ARTs Outcome

After the copula, from the millions of spermatozoa ejaculated, only a small number of about few hundreds make it to the region of the oviduct (ampulla), where they encounter the egg and fertilization occurs (Williams et al., 1993; Hino et al., 2016). Presumably, this subpopulation has been selected through the oviduct in a way that only those with the highest fertilization capability and the best features for supporting embryo development get the opportunity to fertilize the egg (Sakkas et al., 2015; Pérez-Cerezales et al., 2017). Thus, it has been proposed that one of the reasons for the relatively low efficiency of ARTs is that we currently lack an effective methodology to separate this specific sperm subpopulation for its use in ARTs (Sakkas et al., 2015; Pérez-Cerezales et al., 2017). This is especially relevant if we consider that both IVF and ICSI bypass the sperm selection operating *in vivo*, increasing the risk of fertilizing the oocyte with defective spermatozoa that could lead to developmental failure and even affect the offspring in the long run (Fernandez-Gonzalez et al., 2008). This risk could be higher in the clinical practice since the incidence of sperm abnormalities, including DNA fragmentation, is higher in infertile men (Saleh et al., 2003; Ozmen et al., 2007; Schulte et al., 2010).

The extent to which the initial quality of the sperm sample affects ARTs success is not clear. It has been generally accepted that depending on the nature of the male factor, IVF or ICSI should be followed as a preferable treatment. In contrast to IVF, ICSI bypasses the last barriers that the spermatozoa have to overcome during fertilization, increasing the risk of fertilizing the oocyte with defective spermatozoa. Therefore, the recommendation is to use always IVF as first choice and ICSI only in the following cases: testicular and epididymal spermatozoa, immotile but viable spermatozoa, asthenozoospermia, globozoospermia, teratozoospermia, and with frozen spermatozoa (Tournaye, 2012). However, because ICSI has been proven to report the same efficiencies as IVF (Schwarze et al., 2017), nowadays this is the most used technique in fertility treatments in developed countries, replacing IVF as the first choice (71.3 and 72% of total cycles were performed by ICSI in Europe in 2014 and in United States in 2016, respectively) (CDC, 2018; De Geyter et al., 2018). This goes against the effectiveness of the last spermatozoa selection barriers imposed by the oocyte during IVF. In a former study, Jones et al. (1998) reported a negative correlation between the presence of male infertility factor and the IVF outcome in terms of blastocyst production. However, in this study and another one conducted by Boulet et al. (2015), no differences were found in final ratios of live births among patients with or without male factor using IVF or ICSI (Jones et al., 1998; Boulet et al., 2015). Recently, Chapuis et al. (2017) reported that using ICSI, patients showing severe oligospermia showed reduced blastulation rate, whereas a reduced progressive motility affected the fertilization and cleavage rates when IVF was performed. Pregnancy rates also decreased in both IVF and ICSI when the father was older than 51, although female age could also have conditioned these results.

Chromatin integrity is the most studied parameter out of the classical and routinely evaluated features of spermatozoa. However, its power to predict ARTs outcome is also under discussion and there are a number of publications showing opposite results as reviewed by Schulte et al. (2010). Latter reviews including a meta-analysis from existing literature corroborate that abnormal chromatin is associated to male infertility and suggest a negative correlation with the probability of ARTs to succeed (Simon et al., 2017b, c). Recent works proved that sperm DNA fragmentation delays embryo cleavage when donated oocytes are used for ICSI but does not affect the final quality of the embryo (Esbert et al., 2018; Casanovas et al., 2019). Also recently, Jerre et al. (2019) retrospectively analyzed the use of semen with different degrees of chromatin packaging for IVF and ICSI in 1602 pregnancies and found that, above certain threshold, spermatozoa porting immature chromatin slightly increased the risk of early miscarriage. The contradictory results reported in the literature could be related to the different methods used for evaluating genetic integrity, all susceptible of variability between different labs. For further insight into the principles of the methods employed for the determination of chromatin integrity, their advantages and disadvantages, and their correlation with male infertility, we address the reader to specific reviews available in the literature (Raheem and Walsh, 2016; Majzoub et al., 2019).

The difficulties to establish a clear correlation among the presence of male factor, sperm chromatin status, and ARTs success could be due to the multifactorial nature of fertility, where the oocyte, spermatozoid, endometrial environment, etc., are individually determinant on pregnancy failure. An under-evaluation of the sperm quality could also be withholding this correlation, since different features are currently not taken into consideration in andrology laboratories as a routine, due to the complexity of the required analysis and to the lack of basic research on molecular markers of sperm quality. Recent studies have also revealed the complex composition of the ejaculate at different levels including epigenetics, indicating that a number of sperm subpopulations with a wide diversity of features coexist (see the next section). Thus, further research should be done to identify additional markers in the spermatozoa related to ARTs success that could serve to isolate the sperm subpopulation whose features support a better embryo development and pregnancy to term.

### Sperm Heterogeneity

Any given sperm sample shows a complex heterogeneity revealed at different levels. The most obvious intra-sample diversity can be directly seen under the bright field microscope as different motions are shown by each spermatozoon. A closest examination also reveals a mosaic of subpopulations regarding various morphological features. This heterogeneity of the sperm motility and morphology is objectively confirmed by automatized techniques of sperm tracking and analysis (i.e., CASA and CAMA) that classify the spermatozoa into different subpopulations according to specific kinetical parameters (Martínez-Pastor et al., 2011) and morphologies (Yániz et al., 2015; Soler et al., 2016). Moreover, the composition of sperm subpopulations regarding motility is dynamic throughout time. For instance, *in vitro* conditions for capacitation cause time-dependent changes in the spermatozoa at the cellular level that can be recorded as changes in motility, such as the acquisition of the hyperactive motility. This motility type involves vigorous movements under low-viscous conditions produced by asymmetrical and high-amplitude waves in the flagella and resulting in erratic swimming trajectories (Suarez, 2008). However, not all the spermatozoa acquire the hyperactive motility under capacitation conditions, reaching a maximum of around 20% in humans (Burkman, 1984). Furthermore, human spermatozoa also show a heterogeneous response to chemical inductors of hyperactivation. Ooi et al. (2014), following a single cell analysis by high-speed video recording of adhered human spermatozoa, showed that in response to the potent hyperactivation inducer 4-aminopyridine (4AP), multiple patterns of flagellar beating are produced in each spermatozoa, configuring complex and heterogeneous responses to this type of stimulus. Moreover, using the chemoattractant progesterone, another inductor of hyperactivation, Armon and Eisenbach (2011) showed a variety of responses of individual spermatozoa within few seconds immediately after the sperm–progesterone interaction. These observations suggest the existence of different sperm subpopulations, each with a specific sensitivity to respond by chemotaxis, a mechanism leading the spermatozoa to orient their swimming within a chemical gradient (Eisenbach, 1999). In accordance, only 2–12% of the human spermatozoa are able to align the direction of their swimming toward follicular fluid in a chemotactical response (Cohen-Dayag et al., 1994) and only ∼5% of human spermatozoa migrate toward the warmer temperature in response to thermotaxis (Bahat et al., 2012), a mechanism leading the spermatozoa to orient their swimming within a temperature gradient (Bahat et al., 2003).

Sperm heterogeneity has been reported at every level and it can be noticed almost in any study and analysis. Accordingly, a number of research studies hypothesize that only a small subpopulation of spermatozoa within the ejaculate retain the ability to achieve fertilization (Holt and Fazeli, 2015; Sakkas et al., 2015; Pérez-Cerezales et al., 2017). This determines that a high number of spermatozoa are needed to be placed directly onto the oocyte for achieving fertilization *in vitro*. The standard ratio for IVF in humans is 50,000 or more motile sperm per oocyte (Stephens et al., 2013); however, the lower limits of this ratio for achieving fertilization depend on the sperm quality. For example, in a study with subfertile male patients showing diverse sperm qualities, the use of 5000 sperm/oocyte resulted in 37% of zygotes from total oocytes, a percentage that was significantly increased to 60% when using 20,000 sperm/oocyte (Tournaye et al., 2002). The meta-analysis conducted on available publications reporting randomized controlled trials for the treatment of male subfertility evidences a large variability of fertilization ratios, ranging from 50,000 to 10 × 10^6^ sperm/oocyte for IVF (Tournaye et al., 2002). In mice, this limit was reduced to 5 motile sperm/oocyte achieving 60% of fertilization by prolonging the capacitated status of the spermatozoa via creatine supplementation of the medium (Umehara et al., 2018). Therefore, these results also indicate a transient availability of capacitated and “fertile spermatozoa” within the semen. Overall, these data indicate that from the whole spermatozoa population, only a small fraction is susceptible of acquiring the capacity to achieve fertilization at a given time point under *in vitro* conditions.

This observable intra-sample heterogeneity is surely determined by underlying differences among spermatozoa at the cellular and molecular levels. Using discontinuous Percoll gradient, Buffone et al. (2004) found at the different fractions (45, 65, and 90% of Percoll) that human spermatozoa possessed different characteristics regarding quality (motility and morphology) and capacity to show hyperactive motility and protein tyrosine phosphorylation in response to incubation under capacitating conditions. A recent study on bull sperm reported that within two different sperm populations separated by density gradient centrifugation (DGC), there were 31 proteins more abundant in low motile spermatozoa, while 80 proteins were more abundant in the high motile population (D’Amours et al., 2018). Following discontinuous gradient centrifugation of spermatozoa from normozoospermic men, Jenkins et al. (2014) proved the existence of differentially methylated regions in the DNA between high-quality and low-quality fractions. Immunocytological studies on the location of opsins in human and mouse spermatozoa revealed heterogeneous staining patterns for each of the studied proteins involved in the thermotaxis response (Pérez-Cerezales et al., 2015) and reported that a specific subpopulation (∼15% of the ejaculate) showing rhodopsin at a specific cellular location is the one with the capability of migrating toward the higher temperature within a gradient (Pérez-Cerezales et al., 2018).

The heterogeneity of the spermatozoa is also reflected at the genetic and epigenetic levels. Thus, single cell analysis of DNA fragmentation by the Comet assay reveals high intra-sample heterogeneity in human spermatozoa, as described by Simon et al. (2017a). We have observed as well that the level of DNA fragmentation of individual spermatozoa ranged from 0 to 70% in normozoospermic men and from 0 to 65% in epididymal mouse spermatozoa (Pérez-Cerezales et al., 2018). The sperm sample also shows various subpopulations at the chromatin packaging level, as evaluated by the sperm chromatin structure assay (SCSA). This assay discriminates subpopulations with high and low chromatin compaction, with different degrees among individual spermatozoids (Evenson, 2016). Moreover, about 9% of the human spermatozoa port some chromosomic alteration (Martin, 2008) and a high percentage of them carries punctual mutations (Wang et al., 2012). In addition, single cell analysis of the telomere length of human spermatozoa also revealed intra-sample heterogeneity with no differences between normal or abnormal spermatozoa (Antunes et al., 2015). These new findings included in the –omics fields reveal the high level of complexity of the ejaculate and the intimate association between these features and male infertility (for a comprehensive review on sperm –omics, we direct the reader to Sinha et al., 2017). The deep study of the genome, epigenome, transcriptome, proteome, and metabolome at the single cell level or in specific sperm subpopulations is expected to reveal higher levels of complexity than the currently known. This type of analyses could help to identify and correlate the presence/absence of specific sperm subpopulations with fertility, and these subpopulations could also be selected for improving ARTs outcome.

In conclusion, it is widely accepted that not all the spermatozoa from an ejaculate are equally good for achieving fertilization *in vivo* or *in vitro* (Holt and Fazeli, 2015). Due to the heterogeneity of the ejaculate, sperm selection prior to ARTs has been considered an important step for ensuring a successful outcome and a strategy to improve ARTs efficiency.

## Routine Preparation of Semen for ARTs

Sperm selection/preparation techniques should be operatively simple and economic in order to fit in the routines of the human and veterinary clinics. Also, they always must ensure the enrichment of the sample in high-quality spermatozoa in the shortest time possible. Besides removing low quality spermatozoa, including those immotile, sperm preparation techniques should allow to eliminate other cells such as leukocytes and bacteria, as well as toxic or bioactive substances like reactive oxygen species (ROS) (Henkel and Schill, 2003).

**FIGURE 1:**
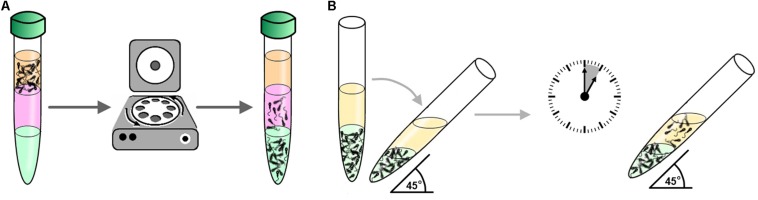
Methods for routine sperm preparation for ARTs. **(A)** Example of sperm selection by density gradient centrifugation (DGC): Semen is placed on top a gradient of colloid (e.g., silica particles coated with silane) prepared in a centrifuge tube and then subject to centrifugation. Subsequently, motile spermatozoa are recovered from the tube bottom containing the fraction with higher density of colloid. **(B)** Example of sperm selection by swim-up (SU): In a centrifuge tube, a medium free of cells (yellow in the figure) is placed on top of the seminal sample (green in the figure). The tube is then inclined 45° and incubated for about 1 h allowing motile spermatozoa to swim-up towards the medium free of cells where they are collected for downstream applications.

Because they satisfy all these requirements, swim-up (SU) and DGC (Figure 1) are the most extended techniques for the preparation of spermatozoa (Henkel and Schill, 2003). SU was first described by Mahadevan and Baker (1984) and the general principle is the recovery of motile spermatozoa that migrate toward a cells-free medium usually placed above the sperm sample. Different variants of SU are available involving centrifugation (Mahadevan and Baker, 1984), straight migration from unprocessed semen (Aitken and Clarkson, 1988), recovery of spermatozoa from non-resuspended pellet (Carreras et al., 1990), or spermatozoa sedimentation by gravity prior to SU [migration sedimentation (MS) method] (Kiratli et al., 2018). On the other hand, DGC is based on the capacity of motile spermatozoa to progress through a gradient of density constituted by colloidal particles during centrifugation (Henkel and Schill, 2003). Variants of the method consist in different types of gradient: continuous or discontinuous (Henkel and Schill, 2003) and substances used to generate the gradient of density: ficoll (Harrison, 1976), PVP-coated silica particles or Percoll, or the current commercial variants such as IxaPrep^®^ (MediCult, Copenhagen, Denmark), SilSelect^®^ (FertiPro N.V., Beernem, Belgium), PureSperm^®^ (NidaCon Laboratories AB, Gothenburg, Sweden), or ISolate^®^ (Irvine Scientific, Santa Ana, CA, United States) (Henkel and Schill, 2003) used in human clinics to substitute Percoll due to its toxicity (Mousset-Siméon et al., 2004) and side effects on the sperm function (Strehler et al., 1998). In all the SU and DGC variants, it has been shown that the recovered sample is enriched in motile spermatozoa showing normal morphology. It has even been observed that both techniques select those spermatozoa with the longest telomeres (Zhao et al., 2016), this being an indicator of correct spermatogenesis (Rocca et al., 2016).

A number of studies have tried to clarify which of these two methods is more efficient; however, the results available in the literature are multiple and contradictory. In a first study approaching sperm selection for ICSI in patients with cryptozoospermia or severe oligoasthenoteratozoospermia (OAT), Sanchez et al. (1996) showed that the MS method (a type of SU) yielded a sperm fraction with better motility, vitality, morphology, and chromatin condensation than when using minipercoll (a type of DGC), achieving 39.7% of pregnancies (*n* = 159 cycles). Also, Zini et al. (2000) showed that DGC is less efficient than SU for the separation out of spermatozoa with less DNA integrity. These results were contradictory to those reported by Sakkas et al. (2000) indicating the opposite. Amiri et al. (2012) reported that spermatozoa from normozoospermic donors selected by DGC showed less DNA fragmentation, higher normal morphology, and higher motility than when selected by SU (24 vs 32%, 28 vs 14%, and 76 vs 52%, respectively). Supporting these results, Wang et al. (2014) proved that DGC reduces DNA fragmentation in patients with oligozoospermia and astenozoospermia. Karamahmutoglu et al. (2014) reported that the use of spermatozoa of subfertile patients selected by DCG for intrauterine insemination significantly improved the pregnancy rate compared to the use of SU (18 vs 7%). In contrast, Oguz et al. (2018) reported that SU enriched the sample in spermatozoa with less DNA fragmentation in patients with mild or idiopathic male factor, while DGC did not allow such enrichment in those same patients. A variant of SU, the direct micro-SU, has shown comparable fertilization percentages by ICSI to those of DCG but higher blastocyst development *in vitro* and pregnancy rates (42 vs 26%, respectively), also reducing the abortion rate (13 vs 29%, respectively) (Palini et al., 2017).

In livestock species, SU and DGC are also the most used techniques for sperm selection (Arias et al., 2017) and although Percoll is currently avoided in human ART because of safety issues, as above indicated, it is still a method of choice for animal ART (Cesari et al., 2006). In rams, it has been reported that SU gives better results in terms of reducing the presence of apoptotic spermatozoa vs DGC with Percoll (67 vs 72%, respectively). However, DGC with Percoll delivered a higher percentage of capacitated spermatozoa than the SU method (Martí et al., 2006). In bovine, when comparing DGC (BoviPure^TM^) (Nidacon, Sweden) vs SU for selecting frozen-thawed spermatozoa, BoviPure^TM^ resulted in spermatozoa showing a greater progressive motility and viability, as well as a higher blastocyst yield (31.79 vs 21.91%, respectively) (Samardzija et al., 2006). Arias et al. (2017) reported that Percoll gradient recovered more spermatozoa with intact plasma and acrosomal membranes (89.8 and 87.5%) than BoviPure gradient (83.3 and 80.4%), but ROS levels were higher with Percoll separation. In addition, Percoll gradient resulted in lower blastocyst yield in comparison to BoviPure^TM^ (∼20 vs 30%, respectively) using frozen-thawed semen (Lee et al., 2009). Thus, BoviPure^TM^ should be considered the best frozen-thawed sperm selection technique for *in vitro* production of bovine embryos.

Both SU and DGC are regularly used in laboratories around the world prior to IVF and ICSI but, as discussed in the section “Introduction,” the success rates of both techniques should be improved in both human and veterinary practices. Both methodologies for sperm selection are exclusively based on the motile capacity of sperm, which does not mean that all motile sperm are of the highest quality. For instance, it has been pointed that both SU and DGC are not efficient methods to select spermatozoa in terms of apoptosis, DNA integrity, membrane maturation, and sperm ultrastructure (Said and Land, 2011). Furthermore, centrifugation steps inherent to both techniques generate ROS, which have a detrimental effect on sperm quality (Aitken and Clarkson, 1988; Henkel and Schill, 2003). Therefore, new sperm selection methods should be based on sperm characteristics that are better connected to fertilization ability and quality of the spermatozoa, as well as on their contribution to support embryo development to term.

## Direct Selection of Immotile Sperm

It is important to note that samples in which spermatozoa are immotile, or where the percentage of motile spermatozoa is low, are not suitable for SU and DGC methodologies. This is the case of sperm samples obtained by testicular sperm extraction (TESE) in azoospermic (AO) patients. Nowadays, in the routine practice of fertility clinics, only vague and subjective morphological criteria are followed to select the immotile spermatozoa prior to ICSI, like the identification of spermatozoa with normal head and tail. However, in the last decades, a number of efforts have been made in order to find a selection method for this type of sample, aiming to discriminate viable spermatozoa directly under the micromanipulator irrespectively of their motility.

Marques de Oliveira et al. (2004) proposed the mechanical touch technique (MTT). According to these authors, MTT allows to identify immotile but viable spermatozoa because their tail is flexible when applying a lateral force to the flagella with the ICSI micropipette. In contrast, flagella of non-viable spermatozoa remain rigid to the same force. These authors conducted the only published clinical trial of MTT and found no differences in the ICSI outcome, including deliveries, between motile spermatozoa, and those immotile selected by MTT, in both fresh or frozen samples (Marques de Oliveira et al., 2004). Unfortunately, this study was never corroborated and completed by others. It would be desirable to report as well the comparison between the use of viable vs non-viable spermatozoa selected by MTT for ICSI. Without this information, it is difficult to envisage the actual utility of this technique.

Another sperm selection method that has been investigated is the hipo-osmotic swelling (HOS) test. This technique is based on the fundament that the tails of viable spermatozoa swell or curl under hypo-osmotic conditions due to normal membrane function, thus allowing their identification and recovery under the microscope, potentially for their use for ICSI (Verheyen et al., 1997). Sallam et al. (2005) conducted a randomized study with 79 couples following TESE-ICSI. In this study, where both frozen and fresh samples with total absence of sperm motility were used, pregnancy rates were 20.5% for the 44 couples following HOS and 2.9% for the 35 couples following morphological evaluation (normal head and tail) prior to ICSI. Fertilization and grade I and II embryos rates were also significantly higher in the HOS group. However, when data were analyzed separately, considering the type of sperm sample (fresh or frozen), pregnancy results were not significantly different.

Under polarized light microscopy, the head of viable spermatozoa is birefringent. On this basis, Baccetti (2004) proposed to use this property as a parameter for sperm selection. Indeed, the selection of immotile spermatozoa with birefringent head for ICSI has shown to increase clinical pregnancy and implantation rates compared to control immotile spermatozoa (58 vs 9% and 42 vs 12%, respectively) (Gianaroli et al., 2008) and to increase clinical pregnancy when compared to those selected by HOS (45 vs 11%, respectively) (Ghosh et al., 2012).

The use of chemical inducers of sperm motility has also been investigated. Thus, phosphodiesterase inhibitors of the xanthine family, such as pentoxifylline (PTF), demethylxanthine theophylline (TPF), and papverine have shown to activate the motility in a fraction of immotile testicular sperm (Taşdemir et al., 1998; Ebner et al., 2011; Terriou et al., 2015). The use of this motile fraction, whether using PTF, TPF, or papverine, has allowed to achieve normal fertilization, pregnancy, and birth after ICSI (Terriou et al., 2000; Kovačič et al., 2006; Amer et al., 2013; Sandi-Monroy et al., 2019). Furthermore, Mangoli et al. (2011) showed that the use of PTF compared with HOS selected spermatozoa, yielded higher fertilization rate, and doubled clinical pregnancies (32 and 16%, respectively, *n* = 25). Interestingly, the successful treatment of male infertility associated to the Kartagener’s syndrome has been reported with both PTF and HOS, achieving the delivery of healthy babies in ICSI cycles (Hattori et al., 2011; Montjean et al., 2015). These findings show the therapeutic potential of both methodologies that could be useful for the treatment of other male infertility cases.

Motility of immotile but vital spermatozoa can also be induced with a single laser shot to the tip of the flagellum (Aktan et al., 2004). Thus, the laser-assisted immotile sperm selection (LAISS) has been proposed as an alternative to the use of chemicals like xanthines, avoiding in this way their potential toxic effects. This method has shown to significantly increase cleavage and birth rates after ICSI using both testicular and ejaculated sperm when compared to control groups (Aktan et al., 2004; Nordhoff et al., 2013). Moreover, LAISS has shown its utility to restore fertility following ICSI in specific cases of male patients showing primary cilia dyskinesia (Gerber et al., 2008) and the Kartagener’s syndrome (Ozkavukcu et al., 2018). Despite the potential of this technique, its complexity and cost have possibly prevented its use in clinical routines and explain the scarce number of published studies.

In addition to these specific procedures, the methodologies described in the following sections, that do not require sperm motility, in principle are also suitable for sperm selection in immotile samples, although their efficiency for this type of samples should be further explored.

## Sperm Selection Based on Membrane Characteristics

The outer membrane of the spermatozoa is crucial for their functionality as it takes part in a number of basic processes such as cell metabolism, capacitation, ova binding, acrosome reaction, etc. Its accessibility and relation with sperm vitality and quality make this organelle, its integrity, and variable characteristics, a logic target for the development of selection methods of high-quality spermatozoa for their use in ARTs.

### Annexin V Magnetic Activated Cell Sorting (AV-MACS)

**FIGURE 2:**
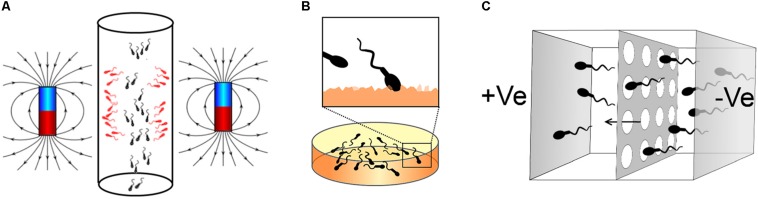
Methods for sperm selection based on membrane characteristics. **(A)** Example of Annexin V magnetic activated cell sorting (AV-MACS): Apoptotic spermatozoa (red in the figure) are bound to magnetic nanoparticles coated with Annexin V and affinity to the externalized phosphatidylserine in this sperm population. Semen is then passed through a column with magnetos on the side, thus apoptotic spermatozoa are retained and non-apoptotic are washed out for downstream applications. **(B)** Example of method for sperm selection based on hyaluronic acid-binding. Spermatozoa are placed on a dish coated with hyaluronic acid (HA, orange in the figure). Matured spermatozoa bind to the surface due to their interaction with the HA. These spermatozoa can be then recovered for ICSI using a micromanipulation system. **(C)** Example of sperm selection by the Z-method developed by Ainsworth et al. (2005): Spermatozoa are placed in a well isolated from another adjacent well by a porous membrane and filled with a medium free of cells. An electric field is then applied with the anode in the well free of cells and the cathode in the well containing the sperm suspension. Due to the negative charge of the sperm membrane, spermatozoa migrate towards the compartment free of cells passing through the porous membrane. Spermatozoa can be then collected for downstream applications.

Magnetic activated cell sorting (MACS) is a method that allows the separation of cell populations based on their surface antigens (Plouffe et al., 2015). Coating magnetic nanoparticles with a molecule with affinity for these antigens allows the trapping of the desired cell subpopulation and its separation within a column subject to a strong magnetic field. Thus, cells expressing the antigen stay in the column while other cells flow through for downstream applications. The loss of membrane integrity is an early event of the apoptotic response and leads to the externalization of phosphatidylserine (Elmore, 2007). On this basis, magnetic nanoparticles coated with Annexin V, a molecule showing high affinity for phosphatidylserine (Vermes et al., 1995), bind to apoptotic spermatozoa that are retained in the MACS column, allowing the recovery of non-apoptotic spermatozoa for their use in ARTs (Gil et al., 2013) (Figure 2A).

The use of Annexin V MACS (AV-MACS) for sperm selection in the human clinical practice was first reported by Grunewald et al. (2001), demonstrating the utility of this technique for the enrichment of the sample with non-apoptotic spermatozoa retaining membrane integrity. Since then, a number of studies have shown the enrichment of high-quality spermatozoa with reduced levels of DNA fragmentation using AV-MACS (Table 1). Said et al. (2006) employed AV-MACS for the selection of semen from 35 healthy donors, reporting an enrichment of spermatozoa with lower DNA fragmentation and higher oocyte penetration capacity. Using this method, Zahedi et al. (2013) also reported an enrichment of spermatozoa with lower DNA fragmentation in both fertile and infertile (terato- and asthenozoospermic) patients. Additionally, Lee et al. (2010) and Degheidy et al. (2015) proved that spermatozoa selected by AV-MACS from idiopathic infertile patients and patients diagnosed with varicocele showed lower DNA fragmentation than the original sample and kept intact sperm motility. However, a number of studies comparing AV-MACS with SU and DGC question the actual utility of this technique for the enrichment in spermatozoa with higher quality. Therefore, Tavalaee et al. (2012) found that combined AV-MACS followed by DGC on semen from infertile patients with different etiologies was more efficient at enriching the sample in spermatozoa with lower DNA fragmentation than both procedures autonomously or DGC followed by AV-MACS. Nadalini et al. (2014) obtained better results with DGC followed by SU than with DGC followed by AV-MACS in terms of sperm quality as determined by motility, morphology, and DNA fragmentation. Cakar et al. (2016) did not find significant differences in any of the analysis of sperm quality, including DNA fragmentation, by using AV-MACS, SU, DG, SU/AV-MACS, and DG/AV-MACS, perhaps due to the low sample size of the study. In contrast, Berteli et al. (2017) found a better quality in terms of motility and DNA fragmentation in spermatozoa selected by AV-MACS followed by DCG than with the other combinations tested: DCG and AV-MACS alone or DCG followed by AV-MACS. Similar to these results, Zhang et al. (2018) reported that the combination of DCG followed by AV-MACS in immotile sperm samples resulted in the recovery of a sperm population with lower DNA fragmentation compared to DGC alone. In agreement, Esbert et al. (2017) reported that DGC followed by AV-MACS reduced the number of spermatozoa porting chromosomal abnormalities.

**TABLE 1:** DNA fragmentation of spermatozoa selected by AV-MACS.

**Groups compared**	**DNA fragmentation (%)**	**Type of male donors/patients (*n*)**	**References**
Unsorted	14.4 ± 13.2^a^	Donors (35)	Said et al., 2006
Annexin positive	21 ± 13.6^b^		
Annexin negative	9.7 ± 10.6^c^		

DGC	13.5 ± 5.6	Normozoospermic (60)	Lee et al., 2010
DGC/AV-MACS	9.9 ± 3.6^*⁣**^		

Unsorted	29,72 ± 3.41^a^	Infertile, various etiologies (15)	Tavalaee et al., 2012
DGC	21,27 ± 3.47^b^		
AV-MACS	21,72 ± 3.41^b^		
DGC/AV-MACS	17,63 ± 3.72^c^		
AV-MACS/DGC	15,27 ± 3.49^c^		

Unsorted	17.7 ± 1.6	Fertile (10), infertile (26): terato- and asthenozoospermia	Zahedi et al., 2013
MACS	12.1 ± 1.7^∗^		

Unsorted	3.48 ± 4.54^a^	Infertile, various etiologies (25)	Nadalini et al., 2014
DGC/AV-MACS	2.41 ± 2.72^b^		
DGC/SU	2.1 ± 2.75^c^		

Unsorted	12.43 ± 6.29	Infertile, vaticocele (36)	Degheidy et al., 2015
AV-MACS	9.61 ± 5.62^∗^		

SU	21.4 ± 16.6	Normozoospermic (10)	Cakar et al., 2016
SU/AV-MACS	15 ± 4.9		
DGC	18.6 ± 5.8		
DGC/AV-MACS	21 ± 6.4		
SU	12 ± 16.6	Oligozoospermic (10)	
SU/AV-MACS	9.4 ± 9.9		
DGC	10.6 ± 8.4		
DGC/AV-MACS	8.4 ± 6.3		

Unsorted	24 (9–26)	Normozoospermic (15)	Berteli et al., 2017
DGC	10 (5–16)		
DGC/AV-MACS	6 (3–11)		
AV-MACS/DGC	4 (2–7)		
AV-MACS	8 (6–16)		

Unsorted	9.56 ± 3.39^a^	Asthenozoospermic (16)	Zhang et al., 2018
DGC	5.25 ± 1.61^b^		
DGC/AV-MACS	2.75 ± 1.2^c^		

*DNA fragmentation was analyzed in all cases by TUNEL. Values of % DNA fragmentation are expressed as % ± SEM, SD or (interquartile range). ^∗^*P* < 0.05, ^∗∗∗^*P* < 0.001 and different letters indicate differences between groups (*P* < 0.05).*

Regarding the use of the spermatozoa selected by AV-MACS for ARTs, reported results are scarce and unclear (Table 2). An early study employing 196 oligoasthenozoospermic patients reported an increase of cleavage and pregnancy rates when AV-MACS was applied prior to ICSI in comparison to DGC (Dirican et al., 2008). However, another study registering 237 infertile couples following ICSI with donated oocytes did not find significant differences in terms of embryo quality and in the ratios of fertilization, implantation, pregnancy, and live birth when the semen was cryopreserved and then selected by SU or SU followed by AV-MACS (Romany et al., 2014). García-Ferreyra et al. (2014) reported higher, but not statically significant pregnancy and implantation rates when semen showing basal high DNA fragmentation was selected by AV-MACS followed by DGC, compared to DGC alone in ICSI treatments. These results are supported by those reported by Stimpfel et al. (2018) in couples of teratozoospermic patients and women over 30 years old undergoing ICSI, showing higher quality blastocysts when spermatozoa were selected by DGC/SU followed by AV-MACS, compared to DGC/SU. Recently, Ziarati et al. (2019) reported a significant increase in the percentage of high-quality embryos and clinical pregnancies employing DGC followed by MACS in 80 infertile couples showing male factor and undergoing ICSI when compared to DGC itself.

**TABLE 2:** Reproductive outcomes of ARTs employing spermatozoa selected by AV-MACS.

**Sperm origin**	**Oocyte origin**	**Sperm groups**	**Fertilization rate (%)**	**Cleavage (%)**	**Blastocyst (%)**	**Embryos transferred (*n*)**	**Clinical pregnancy (%)**	**Live birth rate/ET (%)**	**References**
Fresh. oligoastheno- zoospermic	ND	DGC	69.9	88.2	85	ND	36.49	ND	Dirican et al., 2008
		AV-MACS	69.52	97.2^∗^	84.9		48.36^∗^		

Cryopreserved	Donors	SU/AV-MACS	75.3 (71.6–78.9)	ND	ND	123	64.2 (55.4–72.1)	48.4 (39.6–57.1)	Romany et al., 2014
		SU	72.1 (68.6–75.7)			114	71.1 (62.1–78.6)	56.4 (47.3–65.5)	

Fresh. high DNA fragmentation	Donors	DGC	76.5	89.1	41.1	146	45.5	ND	García-Ferreyra et al., 2014
		DGC/AV-MACS	74.5	98.3	50.8	107	63.2		

Fresh. infertile, at least two abnormal parameters	Donors	DGC	74.78 + 3.41	ND	ND	33	24.24	ND	Ziarati et al., 2019
		MACS + DGC	76.13 + 4.38			22	54.54^∗∗^		

Fresh. teratozoospermic	Patients. women aged ≤ 30 years	DGC/SU	70.6	100	56.5	4	50	50	Stimpfel et al., 2018
		DGC/SU/AV-MACS	65.6	95.2	41.2	4	75	50	
	Patients. women aged ≥ 31 years	DGC/SU	79.8	97.5	41.1	5	20	20	Stimpfel et al., 2018
		DGC/SU/AV-MACS	69.5	97	41.4	12	16.7	16.7	

*All the reported cases followed ICSI. Values are expressed as % ± w/o, SEM, SD, or (interquartile range). ^∗^*P* < 0.05, ^∗∗^*P* < 0.01.*

Studies conducted in livestock species using MACS are missing. In rabbits, AV-MACS did not show a clear enrichment in non-apoptotic spermatozoa and did not affect the reproductive outcome when the selected semen was used for AI (Vasicek et al., 2014).

These studies collectively show that the combination of DGC and MACS might provide slight benefits in patients with male factor undergoing ICSI in terms of clinical pregnancy. However, it is not clear to which extent live birth rate is actually improved. Thus, the type of donor or patient and the combination of AV-MACS with other sperm preparation methods could be determinant for improving ARTs. More experiments, registering larger number of patients, and exploring the different variables involved, are needed in order to certificate the utility of AV-MACS for the human clinical practice.

### Hyaluronic Acid (HA) Binding

The hyaluronic acid (HA) is one of the main components of the extracellular matrix surrounding the cumulus-oocyte complex (COC) (Dandekar et al., 1992), and those spermatozoa that follow an adequate spermatogenesis and maturation exhibit binding sites to it (Cayli et al., 2003; Huszar et al., 2006). Therefore, two methodologies of sperm selection have been developed based on the spermatozoa-HA interaction: (i) recovering those spermatozoa trapped on the surface of HA coated dishes (Huszar et al., 2006) (Figure 2B) and (ii) picking up those spermatozoa moving slow when swimming in a medium containing HA in solution (Barak et al., 2001).

According to published data, both methods have shown their capacity to select spermatozoa with lower DNA fragmentation (Table 3). In an early work, Nasr-Esfahani et al. (2008), employing HA-coated dishes, reported a significant inverse correlation among the percentage of HA-bounded spermatozoa and protamine deficiency, DNA fragmentation, and abnormal sperm morphology in the original sample. While Razavi et al. (2010) reported that spermatozoa recovered from HA-coated dishes show the same level of DNA fragmentation than the unselected ones, other laboratories reported higher DNA integrity in spermatozoa selected by HA-binding methods (Parmegiani et al., 2010a; Yagci et al., 2010; Mongkolchaipak and Vutyavanich, 2013) Furthermore, Parmegiani et al. (2010a) and Huang et al. (2015) showed lower DNA fragmentation in spermatozoa selected in HA solution compared to SU, and by HA coated dishes compared to DGC, respectively. However, both studies reported similar levels of DNA fragmentation when compared to spermatozoa selected under the microscope regarding motility and morphological features. Similarly, Mongkolchaipak and Vutyavanich (2013) did not find any difference between DGC/HA coated dishes and DGC/intracytoplasmic morphologically selected sperm injection (IMSI) respect to sperm DNA fragmentation. These results question the utility of sperm selection based on HA-binding.

**TABLE 3:** DNA fragmentation of spermatozoa selected by hyaluronic acid.

**Groups compared**	**DNA fragmentation (%)**	**Type of male donors/patients (*n*)**	**DNA fragmentation technique**	**References**
Unsorted	32.87 ± 8.65	Patients (77): Severe (13%), moderate (61%), and normospermic (26%)	SCD	Razavi et al., 2010
PICSI	30.94 ± 8.7			

Unsorted	16.5^a^	Patients (20): Normozoospermic (12) and oligozoospermic (8)	SCD	Parmegiani et al., 2010a, b
SU	11^b^			
Microscopically selected	11^c^			
Sperm slow	5.3^d^			

Unsorted	45 ± 1.9	Patients (50)	AOF	Yagci et al., 2010
PICSI	0.9 ± 1.9^∗∗^			

DGC	26,8	Donors (50)	TUNEL	Mongkolchaipak and Vutyavanich, 2013
DGC/PICSI	2.6			
DGC/IMSI	1.7			

DGC	33.2^a^	Patients (46)	AOF	Huang et al., 2015
Microscopically selected	17.9^b^			
PICSI	16.1^b^			

*Analysis of DNA fragmentation was conducted by Sperm Chromatin Dispersion (SCD) test, Acridin Orange fluorescence (AOF), or TUNEL. Values of % of DNA fragmentation are expressed as % ± SEM. ^∗∗^*P* < 0.01 and different letters indicate differences between groups (*P* < 0.05).*

Although the use of HA was first conceived for substituting the routinely used PVP as an agent to decrease sperm motion prior ICSI (Sbracia et al., 1997; Barak et al., 2001), the potential as a selective agent was soon explored (Cayli et al., 2003). Thus, Park et al. (2005) used HA solution to select boar spermatozoa and found a significant increase of embryos with normal chromosomal counts and less chromosome abnormalities when compared to conventional ICSI. Few months later, similar results were reported in humans showing that sperm selection by HA led to a reduction of chromosomal disomy frequencies, diploidy, and sex chromosome disomy; all abnormalities associated to embryos produced by ICSI (Jakab et al., 2005). All these publications did not report clear effects on ICSI outcomes; however, this was possibly not carefully studied until 2008. Since then, a number of publications have specifically checked the capability of sperm selection by HA-binding for improving ICSI outcome (Table 4). In a first attempt, Nasr-Esfahani et al. (2008) found an increase of the fertilization rate using HA-coated dishes but there was no detectable effect on pregnancy or implantation rates. Parmegiani et al. (2010b) studied the reproductive outcome in 331 patients undergoing ICSI using HA solution (293 patients) or conventional ICSI (86), finding no differences in clinical pregnancies or live birth ratios but an improvement in embryo quality and implantation rate. In another study, the same authors reported comparable results employing 206 oligozoospermic patients (Parmegiani et al., 2010a). Similar results were reported in subsequent studies using either HA-coated dishes or HA solution (Choe et al., 2012; Parmegiani et al., 2012; Majumdar and Majumdar, 2013; Mokánszki et al., 2014). In contrast, Worrilow et al. (2013) reported that the use of HA-coated dishes significantly increased pregnancy (Worrilow et al., 2013; Mokánszki et al., 2014) and live birth rate (Mokánszki et al., 2014) for those patients undergoing ICSI which semen showed a HA-binding capacity ≤ 65%, as measured by the Hyaluronan binding assay. This effect proves that the method could be useful only in specific cases and indicates the need of a preliminary semen analysis for determining the suitability of the technique. Therefore, Erberelli et al. (2017) reported a significant improvement of ICSI using HA-coated dishes in patients with various categories of male factor. Their results also suggested that the benefit of this method could be higher for teratozoospermic patients. In a randomized study employing a large number of patients (2772 couples), Miller et al. (2019) did not find any significant benefit of HA-coated dishes compared to conventional ICSI and either detected an association between results of Hyaluronan binding assay and ICSI outcomes, contradicting previous works above described. These results collectively show a poor capacity of HA binding methods for improving ICSI; however, more studies are needed to identify the types of patients that could benefit from this procedure.

**TABLE 4:** Reproductive outcomes of ARTs spermatozoa selected by hyaluronic acid.

**Sperm origin**	**Oocyte origin**	**Number of couples**	**Sperm groups**	**Fertilization rate (%)**	**Cleavage (%)**	**Blastocyst (%)**	**Embryos transferred (n)**	**Clinical pregnancy (%)**	**Live birth rate/ET (%)**	**References**
Patients: wide range of sperm quality	Patients. different conditions	50	HA coated dishes	79.4 ± 26	ND	ND	ND	40	ND	Nasr-Esfahani et al., 2008
			PVP	67.7 ± 23.5				55		

Patients: oligozoospermic	Patients: average age of 37–38 years	112	HA solution	91.6	ND	ND	125	24.8	23.2	Parmegiani et al., 2010a
		94	PVP	85.8			105	20.9	18	

Patients	Patients: age ≤ 39 years	293	HA coated dishes	93.4	ND	ND	326	32.8	16.3	Parmegiani et al., 2010b
		86	PVP	87.1			96	21.6	18.8	

Patients: normozoospermic	Patients: ages ranged from 30 to 42 years	18	HA solution	75.7	72.9	22.9	ND	ND	ND	Choe et al., 2012
			PVP	83	83	24				

Patients: motility ≥ 5%, total sperm number ≥ 1 × 10^6^	Patients: age ≤ 40 years	50	HA coated dishes	82	ND	ND	49	42.9	18	Parmegiani et al., 2012
		50	HA solution	82			50	40	15	

Patients	Patients: age < 38 and other selection criteria	71	HA coated dishes	64.7	ND	ND	177	35.2	12.4	Majumdar and Majumdar, 2013
		80	PVP	65.7			192	35	10.9	

Patients	Patients: (age < 40 years)	63	HA coated dishes	ND	ND	ND	ND	50.8^∗^	ND	Worrilow et al., 2013
		58	PVP					37.9		

Patients	Patients (age < 40 years)	102	HA coated dishes	55.7	ND	ND	ND	39.3^∗∗^	0.49^∗∗∗^	Mokánszki et al., 2014
		42	PVP	52.8				26.6	0.27	

Patients: various male factors included	ND	19	HA coated dishes	71.93	95.12	ND	19	42.1^∗∗∗^	ND	Erberelli et al., 2017
		37	PVP	64.14	95.27		37	16.21		

Patients: able to produce freshly ejaculate (ages 18–55 years)	Patients: age (18–43)	1387	HA coated dishes	66	ND	ND	1381	35.2	27.4	Miller et al., 2019
		1385	PVP	69			1371	35.7	25.2	

*All the reported cases followed ICSI. Values are expressed as % ± w/o, SEM, SD, or (interquartile range). ^∗^*P* < 0.05, ^∗∗^*P* < 0.01, ^∗∗∗^*P* < 0.001.*

### Zeta Method

Sperm membrane is negatively charged (Engelmann et al., 1988). On this basis, methods for separating X- and Y-bearing spermatozoa (Engelmann et al., 1988) and for the separation of pure sperm heads from disintegrated mammalian spermatozoa (Chaudhuri and Datta, 1994) were developed decades ago. Later, the methods developed by Chan et al. (2006) and Ainsworth et al. (2005) (Figure 2C) allowed to collect the charged spermatozoa adhered to the wall of a centrifuge tube or migrating within an electric field, respectively. With both methodologies, downstream analysis of the selected spermatozoa revealed an increased percentage of spermatozoa with higher quality and porting high-integrity DNA (Kheirollahi-Kouhestani et al., 2009; Razavi et al., 2010; Zarei-Kheirabadi et al., 2012; Zahedi et al., 2013) (Table 5). This procedure, known as Zeta method, has allowed the selection of spermatozoa with lower DNA fragmentation compared to the HA-coated dish selection (Razavi et al., 2010). Also, sperm DNA fragmentation has been proven to be lower using DGC-Zeta than DGC by itself (Zarei-Kheirabadi et al., 2012) or MACS-DGZ (Zahedi et al., 2013). However, despite these promising results, only one randomized study has been published using spermatozoa selected by Zeta method in patients undergoing ICSI (Esfahani et al., 2016). In this research, significant increases in top quality embryos [45.83 ± 3.11% vs 35.38 ± 4.64% (*P* = 0.04)] and pregnancy rates [39.2 vs 21.8% (*P* = 0.009)] were obtained with DGC/Zeta compared to DGC, respectively. However, further analysis is needed to know the potential of Zeta method for improving ARTs.

**TABLE 5:** DNA fragmentation of spermatozoa selected by Zeta method.

**Groups compared**	**DNA fragmentation (%)**	**Type of male donors/patients (*n*)**	**Fragmentation technique**	**References**
Unsorted	19 ± 0.1	Patients (8)	AOF	Chan et al., 2006
Zeta	11 ± 0.1^∗^			

Unsorted	8 ± 2^∗^	Donors (6)	TUNEL	Ainsworth et al., 2005
Zeta	3 ± 2			

Unsorted	49.65 ± 15.1^a^	Patients (60)	AOF	Kheirollahi-Kouhestani et al., 2009
DGC	32.65 ± 14.38^b^			
Zeta	28.85 ± 15.83^c^			
Unsorted	15.83 ± 8.14^a^	Patients (55)	TUNEL	
DGC	8.17 ± 4.23^b^			
Zeta	5.89 ± 3.92^c^			
Unsorted	50.43 ± 15.58^a^	Patients (51)	SCD	
DGC	33.58 ± 16.24^b^			
Zeta	29.21 ± 14.95^c^			

Unsorted	32.87 ± 8.65^a^	Patients (77), 13, 61, and 26% (severe, moderate, and normospermic respectively)	SCD	Razavi et al., 2010
HA coated dish	30.94 ± 8.7^ab^			
Zeta	18.19 ± 8.64^b^			

Unsorted	21.97 ± 3.84^a^	Patients (30)	TUNEL	Zarei-Kheirabadi et al., 2012
DGC	17.47 ± 3.52^b^			
DGC-Zeta	6.98 ± 1.45^c^			

Unsorted	17.7 ± 1.6^a^	Fertile (10), infertile (26): terato- and asthenozoospermia	TUNEL	Zahedi et al., 2013
MACS-DGC	12.1 ± 1.7^b^			
DGC-Zeta	9.8 ± 1.2^b^			

*Analysis of DNA fragmentation was conducted by Sperm Chromatin Dispersion (SCD) test, Acridin Orange fluorescence (AOF), or TUNEL. Values of % of DNA fragmentation are expressed as % ± SEM or SD. ^∗^*P* < 0.05 and different letters indicate differences between groups (*P* < 0.05).*

### Future of Sperm Selection Based on Membrane Characteristics

As exposed above, sperm selection by AV-MACS and HA does not seem to contribute in general terms to a significant improvement of ARTs outcome, and Zeta method needs to proof its efficiency in larger studies. However, the use of the properties of the plasma membrane for sperm selection could really improve ARTs if markers with higher potential for discriminating high-quality spermatozoa are discovered. Thus, new antigens could be used as targets for selection by MACS, by coated plates or even by fluorescence-activated cell sorting (Funaro et al., 2013). However, this research direction is currently poorly explored, possibly because of the lack of basic knowledge about markers discriminating the various sperm populations contained in the ejaculate. Heidari et al. (2018) explored three surface proteins, HSPA2, Dj-1, and serum amyloid P, as biomarkers of sperm DNA integrity and found that the abundance of these three proteins in the semen was directly correlated to sperm quality, DNA integrity, and embryo quality. Mostek et al. (2018) found that high-quality bull ejaculates showed higher abundance of extracellular sperm surface proteins and that sperm proteins in low-quality ejaculates are characterized by high carbonylation levels. All these membrane features and new ones yet to be discovered could be used with the aim of improving ARTs through sperm selection.

## Sperm Selection Based on Sperm Morphology – Intracytoplasmic Morphologically Selected Sperm Injection (IMSI)

**FIGURE 3:**
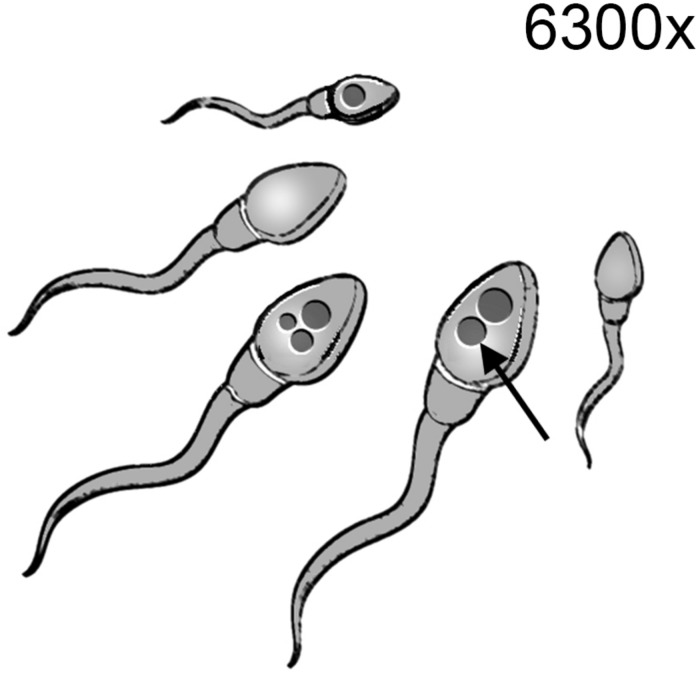
Intracytoplasmic morphologically selected sperm injection (IMSI). Spermatozoa are examined under a magnification system of 6300x in order to discriminate those spermatozoa lacking vacuoles (arrow) and showing normal morphology. These spermatozoa are collected with the micromanipulation system for their use in ICSI.

The morphometric evaluation of spermatozoa is widely applied to analyze sperm quality due to its correlation to fertility (Yániz et al., 2015; Soler et al., 2016). Furthermore, the introduction of computer-enhanced digital microscopy has enabled the analysis and quantification of detailed features of the cell that applied to motile spermatozoa configure the “Motile Sperm Organelle Morphology Examination” (MSOME) (Bartoov et al., 2002). The use of MSOME for selecting spermatozoa for ICSI is known as IMSI. This technique consists in the selection and direct capture of those spermatozoa with a low number of vacuoles and a nucleus of normal morphology under a microscope equipped with a micromanipulation system and a magnification system of 6300x (Bartoov et al., 2002) (Figure 3). The power of selecting spermatozoa with higher DNA integrity has been proved by different authors, although contradictory results are also present in the literature (Table 6). Using this methodology, Franco et al. (2008) revealed that the presence of large vacuoles in the sperm nucleus is associated with a higher DNA fragmentation compared to those spermatozoa with normal nucleus. The correlation between the presence of vacuoles, as detected by MSOME, and DNA fragmentation was later confirmed by Wilding et al. (2011). However, Leandri et al. (2013) did not find significant differences in this respect between the spermatozoa selected by IMSI and those selected as conventionally done during regular ICSI. Other authors have suggested that IMSI could be useful only in specific cases of male infertility. Accordingly, when spermatozoa from infertile donors showing more than 13% of DNA fragmentation were selected by IMSI, those showing normal morphology under high magnification delivered less DNA fragmentation than those classified as motile and normal using conventional magnification of 200x (Hammoud et al., 2013). Moreover, the authors reported in the same work that those selected under lower magnification showed the same DNA fragmentation than the unsorted spermatozoa. Results reported by Lavolpe et al. (2015) suggest that in patients whose semen scored ≤ 4% according to the strict morphology index, the presence of vacuoles in the nucleus of the spermatozoa was less related to DNA fragmentation and chromatin compaction than in those patients with strict morphology index ≥ 14%.

**TABLE 6:** DNA fragmentation of spermatozoa selected by IMSI.

**Groups compared**	**DNA fragmentation (%)**	**Type of male donors/patients (*n*)**	**Fragmentation technique**	**References**
Normal nucleus	15.9//33.1	Patients (30)	TUNEL//AOF	Franco et al., 2008
Large vacuoles	29.1^∗∗∗^//67.9^∗∗∗^			

ICSI	16.2 ± 8.8	Patients: ICSI (139), IMSI (116)	TUNEL	Leandri et al., 2013
IMSI	16.4 ± 9.5			

Unsorted	26.1 ± 1.5^a^	Infertile donors (8) (more than 13% of fragmented DNA spermatozoa)	TUNEL	Hammoud et al., 2013
Motile 200x	20.8 ± 2.7^ab^			
Motile and normal Spermatozoa 200x	18.7 ± 2.7^ab^			
Motile and normal spermatozoa 6300x	4.1 ± 1.1^c^			
Morphometrically normal with anterior/posterior vacuoles 6300x	15.9 ± 2.9^b^/22.5 ± 3.6^ab^			

IMSI no vacuoles	20.1//17.8	Strict morphology index ≤ 4% (26)	AOF//TUNEL	Lavolpe et al., 2015
IMSI vacuoles	22.6−35.2^∗^//18.2−25.4			
IMSI No vacuoles	6.1//3.6	Strict morphology index ≥ 14% (20)	AOF//TUNEL	
IMSI Vacuoles	4.9–36.2^∗^//5.2–16.2^∗^			

*Analyses of DNA fragmentation were conducted by, Acridin Orange fluorescence (AOF) or TUNEL. Values of % of DNA fragmentation are expressed as % ± SEM or SD. Values separated by double slash indicate correspondence to the employed method of DNA fragmentation in that column. In Lavolpe et al. (2015) % of DNA fragmentation are intervals according to different patterns of vacuole number, morphology, and distribution. ^∗^*P* < 0.05, ^∗∗∗^*P* < 0.001 and different letters indicate differences between groups (*P* < 0.05).*

Recently, in the largest study reported to date where 873 sperm samples were analyzed by MSOSE, the presence of head vacuoles was not associated to sperm DNA fragmentation and live birth rate (Fortunato et al., 2016). Authors claim that vacuoles are physiological features that do not alter sperm functionality. The controversy about the convenience of IMSI for improving ARTs exists from the first moment the method was described. We address here the reader to an excellent systematic review on the literature published between 2001 and 2013, where the authors conclude that IMSI was only proven to improve reproductive outcome in cases of recurrent implantation failure following ICSI (Boitrelle et al., 2014). The same year, other authors published a meta-analysis on 13 publications confronting IMSI vs conventional ICSI in cases with previous ICSI failures and when the male factor was the cause of infertility, and concluded that in both cases, IMSI improved the reproductive outcome (Setti et al., 2014). However, authors pointed to the lack of randomized studies to corroborate their conclusions. Since then, together with the study of Fortunato et al. (2016), others have shown the failure of IMSI for improving ARTs (Table 7). Thus, Leandri et al. (2013), having examined 458 couples, did not find significant differences between IMSI or ICSI in the reproductive outcome, regardless of the initial characteristics of semen regarding DNA fragmentation, chromatin compaction, morphology, or motility. Setti et al. (2015) showed no benefits of IMSI for couples with poor ovarian response. In contrast, Shalom-Paz et al. (2015) reported that IMSI significantly increased implantation and clinical pregnancy rates in patients with repeated IVF-ICSI failure. However, Gatimel et al. (2016) failed in confirming these results. Kim et al. (2014) and Goswami et al. (2018) showed that IMSI applied to OAT patients of various severities resulted in significantly higher implantation, pregnancy rates, and live birth rates when compared to a previous ICSI cycle performed to the same patients, suggesting an actual utility of IMSI for these specific cases. Also, in another study on 170 patients, the results suggest that IMSI can improve pregnancy rates in patients with severe sperm pathologies affecting various sperm parameters (Schachter- Safrai et al., 2019). Overall, although the literature dissuades the routine use of IMSI, it seems that it could be highly indicated for the treatment of severe cases of male factor. Larger head-to-head randomized studies discriminating seminal parameters in infertile pathologies are still needed in order to elucidate under which circumstances IMSI could benefit reproductive outcome in the human clinical practice.

**TABLE 7:** Reproductive outcomes of ARTs spermatozoa selected by IMSI.

**Sperm origin**	**Oocyte origin**	**Couples (*n*)**	**Sperm groups**	**Fertilization rate (%)**	**Cleavage%**	**Blastocyst (%)**	**Embryos transferred (*n*)**	**Clinical pregnancy (%)**	**Live birth rate/ET (%)**	**References**
≧3 millions of spermatozoa in the ejaculate, ≦1 million of motile after DGC, whatever sperm morphology	Younger than 39 years and FSH under of 9 UI/L	458	IMSI	56	ND	ND	ND	31	27	Leandri et al., 2013
			ICSI	63				33	30	

Oligo-astheno-teratozoospermia	Patients	66	ICSI	65 ± 21.1	ND	ND	64	6.3	ND	Kim et al., 2014
			IMSI	67.7 ± 19.9			66	27.3		

At least one sperm pathology	Infertile Women	42	IMSI	51.4	95.3	ND	ND	41.3^∗^	34.7^∗∗^	Shalom-Paz et al., 2015
			ICSI	53.2	92			10.5	0	

Patients	Normo response (> 4 oocytes retrieved)	324	ICSI	75.9 ± 18.9	ND	30.9 ± 28.1	ND	39,4	ND	Setti et al., 2015
			IMSI	72.3 ± 21.2		29.3 ± 25.7		34,1		
	Poor response (< four oocytes retrieved)	90	ICSI	79.8 ± 29.3	ND	9.8 ± 21.1	ND	11.8	ND	
			IMSI	53.9 ± 36.7		13.9 ± 33		22.2		

Couples with two previous ICSI failures		216	IMSI	54	ND	ND	119	27	20	Gatimel et al., 2016
			ICSI	61			86	28	19	

OAT/severe OAT (SOAT) or teratozoospermia	Patients	57	IMSI	52^∗∗^	ND	ND	ND	68.5^∗∗∗^	62.4^∗∗∗^	Goswami et al., 2018
			ICSI	30				30.2	0	

Mild to severe oligo, astheno, and/or teratozoospermia	Donors	848	IMSI	82	70	42	511		63,79	Gaspard et al., 2018
			ICSI	72	69	40	923		69,33	

*Values are expressed as % ± w/o, SEM, or SD. ^∗^*P* < 0.05, ^∗∗^*P* < 0.01, ^∗∗∗^*P* < 0.001.*

## Sperm Selection Based on Guidance Mechanisms

As we discussed in the section “Introduction,” guidance within the female genital tract has been suggested as a physiological mechanism for the selection of those spermatozoa able to fertilize the egg and ensuring the ulterior embryo development to term (Pérez-Cerezales et al., 2017). Hence, a strategy to develop new sperm selection methodologies is to employ *in vitro* the same principles that govern the sperm selection operating within the female genital tract. Consequently, there are three known mechanisms that have been proposed to guide the spermatozoa within the oviduct and they have been tested accordingly for their capacity to select spermatozoa with the aim of improving ARTs.

### Rheotaxis

Miki and Clapham (2013) proved for the first time that both human and mice spermatozoa orientate their swimming against a fluid flow, process known as rheotaxis. Latter, El-Sherry et al. (2014) and Romero-Aguirregomezcorta et al. (2018) confirmed the occurrence of this phenomenon also in bovine, stallion, and ram spermatozoa, suggesting rheotaxis as a conserved feature of mammalian spermatozoa. Furthermore, the existence in mice of oviductal flow toward the uterus intensified after copula supports rheotaxis as a long-distance guidance mechanism within the oviduct (Miki and Clapham, 2013).

**FIGURE 4:**
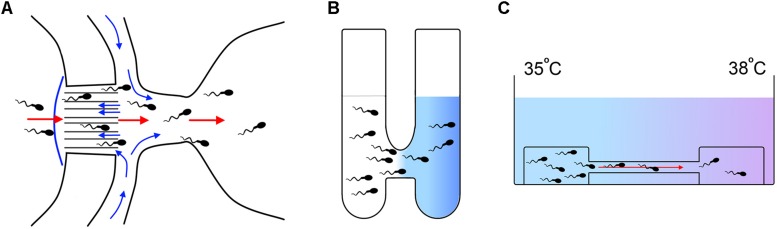
Methods for sperm selection based in guidance mechanisms. **(A)** Method of sperm selection by rheotaxis developed by Nagata et al. (2018). A flow (blue arrows) is created through a serial of microchannels towards a well were sperm sample is placed. In response to the flow the spermatozoa swim towards it passing through the microchannels and accumulating in a receptive well where they can be collected for downstream applications (red arrows indicate migration of the sperm). **(B)** Method of sperm selection by chemotaxis developed by Gatica et al. (2013): Two wells are connected by a 2 mm length per 2.5 mm diameter tube. One of the wells is filled with a medium containing the chemoattractant molecule in solution (colored in blue) and the other well is filled with the spermatozoa suspension. The chemoattractant diffuses through the connecting tube generating a gradient and the spermatozoa respond by migrating towards the higher concentration and accumulating in the initial well free of cells where they can be collected for downstream applications. **(C)** Method of sperm selection by thermotaxis developed by Pérez-Cerezales et al. (2018). Spermatozoa are placed on a drop of a medium connected by a capillary to a second drop free of cells. A temperature gradient is generated between both drops being the highest temperature at the place of the drop free of cells. Spermatozoa respond by thermotaxis migrating towards the warmer temperature and accumulating in the second drop where they can be collected for downstream applications.

De Martin et al. (2017) conducted a first study employing rheotaxis to select spermatozoa from normozoospermic donors and reported an enrichment in spermatozoa with higher chromatin compaction (99%) compared to the sample before selection (71%) and to spermatozoa selected by DCG (83%). Zaferani et al. (2018) reported the design of a microfluidic device for selecting motile spermatozoa on the basis of rheotaxis; however, these authors did not analyze the quality of the selected spermatozoa. Nagata et al. (2018) used another microfluidic device to select frozen bull spermatozoa by rheotaxis (Figure 4A). These authors reported a reduction of the level of DNA fragmentation (0.37%) in the spermatozoa selected by rheotaxis when compared to unselected semen (7%). The use of these selected bull spermatozoa for artificial insemination delivered pregnancy results similar to semen without selection, but using a 20 times lower dose (1 million sperm/insemination against 20 million sperm/insemination, respectively).

Thus, the results reported to date are promising, but the passive nature of rheotaxis, which is based on the hydrodynamics of the motile spermatozoa (Zhang et al., 2016), indicates that this mechanism has poor selective potential beyond the separation of those spermatozoa with a correct swimming behavior. As a matter of fact, half of the spermatozoa orient their swimming by rheotaxis, independently of being or not capacitated when directly observed under the microscope (Miki and Clapham, 2013). Although this is the *in vitro* situation, the *in vivo* picture could be drastically different. Moreover, it has been proposed that the planar move of non-capacitated spermatozoa would make them easier to stick to the epithelium preventing their migration by rheotaxis (Miki and Clapham, 2013) under the physiological conditions of viscosity. In contrast, the rotation along their longitudinal axis that determines the swimming of capacitated spermatozoa under a viscous medium could allow their detachment from this epithelium, allowing their free swimming against the fluid current (Miki and Clapham, 2013). In this way, rheotaxis would be part of a selection system of capacitated spermatozoa allowing only this subpopulation to migrate toward the *in vivo* fertilization site. *In vitro* developed procedures could test this hypothesis for sperm selection by rheotaxis considering these physiological conditions and then configure an effective way of selecting capacitated spermatozoa for improving ARTs.

### Chemotaxis

Chemotaxis is the mechanism of navigation that the spermatozoa use in the proximity of the oocyte to orient their swimming in a gradient of chemical substances released by the COC where progesterone (P4) seems to be the main chemoattractant (Oren-Benaroya et al., 2008). It should be noted that only capacitated spermatozoa respond by chemotaxis, so this property could allow the selection of this specific subpopulation (Oren-Benaroya et al., 2008). Thus, Gatica et al. (2013) selected spermatozoa from both normozoospermic donors and subfertile patients by employing a simple device named “Sperm Selection Assay” (SSA) in which a P4 gradient was established (Figure 4B). In this work, it was found that in both cases, the selected spermatozoa were three times more capacitated, showed less DNA fragmentation and less oxidative stress than the semen before the selection. Li et al. (2018) also reported an improvement in the sperm quality from normozoospermic donors in terms of normal morphology, lower DNA fragmentation, and lower percentage of apoptotic spermatozoa when using another device composed of a system of microfluidic channels. Dominguez et al. (2018) proved that the use of the SSA device allows the selection of higher quality bull spermatozoa, improving cleavage rates using both sexed and unsexed semen for IVF. However, the effectiveness of sperm selection by chemotaxis in the improvement of ARTs in clinical practice has not been studied to date.

### Thermotaxis

Sperm thermotaxis consists in the orientation of the spermatozoa movement toward the highest temperature in a gradient (Bahat et al., 2003). Evidence indicates that this mechanism allows sperm to orient itself in the fallopian tubes to ascend to the ampulla (Pérez-Cerezales et al., 2017). As in chemotaxis, only capacitated spermatozoa respond to thermotactic stimuli, so based on it, this subpopulation could be selected (Bahat et al., 2003). In a recent publication, employing a simple method (Figure 4C), we showed in mice and in normozoospermic patients that spermatozoa selected by SU/thermotaxis possess higher DNA integrity compared to the seminal sample prior to selection and after selection by SU (Pérez-Cerezales et al., 2018). In addition, the use of these selected spermatozoa for ICSI in mice increased cleavage rates and blastocyst production, as well as implantation and live birth rate as opposed to the use of spermatozoa selected by SU. Although this is the only work related to the use of spermatozoa selected by thermotaxis in ARTs, a recent study carried out in bulls has shown that seminal samples delivering high pregnancy rates after artificial insemination show a greater response to thermotaxis (Mondal et al., 2016). However, as in the case of rheotaxis and chemotaxis, there are still no available publications showing the capability of sperm selection by thermotaxis to improve the ARTs efficiency in the human clinic.

## Sperm Selection Based on Sperm Motility (Microfluidics)

As we have seen in some of the previous methodologies, microfluidic systems are becoming transversal devices for sperm selection based on several fundaments. Therefore, microfluidic systems have been tested for sperm selection by rheotaxis (Zaferani et al., 2018), chemotaxis (Ko et al., 2018; Nagata et al., 2018), and thermotaxis (Ko et al., 2018), as well as by other physical properties that operate at small scale, allowing the selection of motile spermatozoa (Table 8). Nosrati et al. (2014) reported the improvement of human spermatozoa selected by a microfluidic device composed of 500 parallel microchannels in terms of reducing more than 80% DNA fragmentation. Similar results were reported by Kishi et al. (2015), showing a significant reduction of DNA fragmentation in spermatozoa selected by a commercially available device based on microfluidics and flow dynamics (Sperm Sorter Qualis^®^, Menicon, Kasugai, Japan) when compared to SU or unselected spermatozoa. Using the same device, Shirota et al. (2016) also reported the selection of spermatozoa with lower DNA fragmentation compared to spermatozoa selected by DGC followed by SU in healthy donors. In addition, Nagata et al. (2018) reported a robust selection of human spermatozoa porting low DNA fragmentation by utilizing a diffuser-type microfluidic sperm sorter (DMSS). Quinn et al. (2018) employed another commercially available device, a single-use chip with an inlet sample chamber connected to an outlet collection chamber by a microfluidic channel (FERTILE, Zymot, DxNow Inc., Gaithersburg, MD, United States), and showed a very effective selection of spermatozoa with low fragmented DNA compared to unselected spermatozoa or those selected by DGC-SU.

**TABLE 8:** DNA fragmentation of spermatozoa selected by microfluidic devices.

**Specie of study**	**Groups compared**	**DNA fragmentation (%)**	**Type of male donors/patients (*n*)**	**Fragmentation technique**	**References**
Human	Control	10.9	Healthy donors (8)	SCSA	Nosrati et al., 2014
	Microfluidic radial 500 channels in parallel	4.32/3.37/2.40			

Human	Control	27.7 ± 0^a^	Donors (10) normal, oligozoospermia, and asthenozoospermia	SCD	Kishi et al., 2015
	SU	8.3 ± 0.05^b^			
	Sperm sorter qualis	5.9 ± 0.04^c^			

Human	DGC-SU	10.1 ± 8.5	Donors (37)	SCSA	Shirota et al., 2016
	Sperm sorter qualis	0.8 ± 1.9^∗^			

Bovine	Unsorted	7.08 ± 1.06	7	TUNEL	Nagata et al., 2018
	Microfluid DMSS	0.37 ± 0.15^∗∗^	12		

Human	Control	15 (11–19)^∗∗∗^	Samples (70)	SCD	Quinn et al., 2018
	DGC-SU	6 (3–11.5)^∗∗∗^			
	Fertile (Zymot) device	0 (0–2.4)			

*Analyses of DNA fragmentation were conducted by sperm chromatin structure assay (SCSA), sperm chromatin dispersion (SCD) test, or TUNEL. Values of % of DNA fragmentation are expressed as % ± SEM or SD. In Nosrati et al. (2014), DNA fragmentation separated by slash corresponds to the microchannel lengths 6/7.5/9 mm, respectively. Negative linear correlation between microchannel lengths was reported (*R*^2^ = 0.99). ^∗^*P* < 0.05, ^∗∗^*P* < 0.01, ^∗∗∗^*P* < 0.001. Different letters indicate differences between groups (*P* < 0.005).*

Due to the novelty of these methodologies, there is only one study that employs the selected spermatozoa in ARTs. In a recent publication in which spermatozoa were selected from 122 patients with infertility of unknown etiology using a commercial microfluidic device (61 patients) (Fertile Chip^®^, KOEK Biotechnology, Turkey) or by SU (61 patients), no differences were found in terms of fertilization, pregnancy, and live birth rates during ICSI between both sperm selection methods (Yetkinel et al., 2019). However, these authors observed a higher quality in those embryos produced with spermatozoa selected by Fertile Chip^®^. Further studies are then necessary to determine under which conditions these devices are suitable for improving ARTs outcomes.

## Conclusion

The efficiency of ARTs has a margin for improvement. Sperm selection may be an important factor for achieving higher live birth rates in ARTs, especially in infertility cases where the male factor is present. However, the methodologies developed to date have not proven to be useful for their routine application in the clinical practice and seem to be effective only in specific cases of male infertility. Some novel methods described here based on the physiological selection operating *in vivo* and on microfluidic environments have delivered promising results yet to be confirmed in large studies in the context of clinical practice. These studies should be randomized and strict in the confrontation of results with sperm samples of different qualities minimizing the female factor. It may even be necessary to combine several sperm selection methodologies to increase the efficiency of the ARTs.

## Author Contributions

IO-L and SR-D conducted literature review and wrote the manuscript. PR-I wrote the manuscript. SP-C defined the topic, conducted literature review, and structured and wrote the manuscript.

## Conflict of Interest

The authors declare that the research was conducted in the absence of any commercial or financial relationships that could be construed as a potential conflict of interest.
